# Close to a Decade of Decrease in Antimicrobial Usage in Danish Pig Production–Evaluating the Effect of the Yellow Card Scheme

**DOI:** 10.3389/fvets.2020.00109

**Published:** 2020-03-06

**Authors:** Ana Carolina Lopes Antunes, Vibeke Frøkjær Jensen

**Affiliations:** Division for Diagnostics & Scientific Advice—Epidemiology, Center for Diagnostics, National Veterinary Institute, Technical University of Denmark, Lyngby, Denmark

**Keywords:** antimicrobials, Yellow Card scheme, legislation, pig, Denmark

## Abstract

The emergence and dissemination of antimicrobial resistant pathogens have led to rising concern about the widespread and excessive use of antimicrobials in veterinary medicine. Denmark has implemented several initiatives to reduce antimicrobial use in animals since the 90s, with the Yellow Card scheme implemented in 2010. The aim of the present study is to investigate the effects on antimicrobial use in Danish pig farms of changes in the legislation regarding the Yellow Card, based on analysis of temporal trends in monthly antimicrobial consumption calculated at herd-level from 2010 to 2017. A dynamic linear model with a linear growth component was used to model the data. The percentage of herds with a negative growth component and a significant decline below zero (based on 95% credible intervals) was assessed for the entire study period. The analysis was run separately for the different age groups within each farm: weaners (up to 30 kg), sows and finishers herds, and categorized according to antimicrobial use before the Yellow Card initiative (2008–2009), as groups of herds with “low,” “moderate,” or “high” consumption. The results evidence a decline of the antimicrobial consumption in Danish pig herds, more pronounced during two periods: with the introduction of the Yellow Card and in relation to the announcements and changes in the legislation in 2014. Weaner herds in the high consumption category had the highest percentage of herds with a negative growth component, reaching 82% of herds in January 2011, followed by weaner herds with moderate antimicrobial consumption reaching 71% of herds in October 2012. For finisher herds in the moderate consumption category, the proportion with a negative growth increased from 38% in January 2010 to 71% in July 2011. A decline in antimicrobial consumption was also evident after announcements and changes in the legislation in 2014, particularly for sows and finishers. Our findings suggest that changes in the Yellow Card scheme promoted a continuous reduction in antimicrobial use in Danish pig herds, particularly in herds with high antimicrobial consumption.

## Introduction

The emergence and dissemination of antimicrobial resistant pathogens have led to rising concern about the widespread and excessive use of antimicrobials in veterinary medicine. Since the 90s, Denmark has implemented multiple initiatives to reduce antimicrobial use in animals, such as the legal ban of the growth-promoters (avoparcin in 1994 and avilamycin in 1996), the restriction of veterinarians' profit from sales of prescription medicines since 1994, and a voluntary food industry ban of all antimicrobial growth-promoters by 1999 (1). In 2001, the Danish Veterinary and Food Administration (DVFA) established a database, recording and monitoring the use of prescribed drugs in animals: the VetStat (2). VetStat has continuously collected detailed data on antimicrobial prescription and sales for livestock farms. These data have been used to generate national summary statistics of the amount and type of antimicrobial prescribed for different animal species in Denmark and to generate research, both supporting the interventions to promote prudent use (1, 3). According to Danish legislation, the use of antimicrobials in animals is only allowed for the treatment of infectious diseases and not for prophylaxis or growth promotion purposes (4).

Currently, Denmark is one of the largest pig producing countries in the European Union with a substantial pig production compared to its relatively small human population. In 2017 (3rd quarter), Denmark had 3,226 pig farms with 12.69 million animals (5) compared to a human population of 5.76 million individuals (6). The vast majority of the pig production is exported, either as live pigs (42.5% of the animals produced in 2017) or pork. Danish pig farms account for 74% of the total veterinary antimicrobial consumption measured in kg of active antimicrobial ingredients in 2017 (3). Furthermore, pigs were also among the animal species receiving the most antimicrobial per kg live biomass (3). During the last decade, the antimicrobial use for pigs has decreased almost continuously since 2009 (7). Within this period, the major intervention by the authorities was the Yellow Card scheme to target pig farmers using high amounts of antimicrobials per animal. Yellow Cards are issued to farmers exceeding usage thresholds, which are dynamic and are defined in the legislation. Initially, in 2010, the thresholds were set at twice the average use in herds of any of the three age-groups: weaners (7–30 kg), sows (including gilts, boars, and piglets) and finisher herds (>30 kg—slaughter). In June and July 2010, farmers with herds above the 80th percentile received a letter with a warning for their proximity to thresholds, and the Yellow Card legislation was implemented in December 2010, issuing Yellow Card from August 2011 onwards. A Yellow Card releases an order to reduce antimicrobial usage below the threshold within 9 months. If this target is not reached, a strategy for reduction is defined with a second-opinion veterinarian, which might include vaccination protocols, management changes, etc., and quarterly visits from the authority. The farmer covers all costs. If database errors are the cause of seemingly high levels for antimicrobial usage or if the high level of antimicrobial usage could be justified (e.g., by a major disease outbreak), the Yellow Card could be withdrawn by the authorities. National statistics on antimicrobial usage have suggested that the Yellow card has been driving a decrease in antimicrobial usage in pig production (3, 8).

However, to date, no published studies have substantiated the effect of the Yellow Card Scheme by comparison of the antimicrobial consumption in Danish pig farms-level with changes in Yellow card Scheme legislation. This information is important for DVFA and the Danish pig industry to evaluate the implementation of the Yellow Card Scheme and to understand when farmers and veterinarians react to changes in the legislation. Additionally, this information could be useful to other countries considering initiatives to reduce antimicrobial consumption. The aim of the present study is to investigate the effects of changes in the legislation regarding the Yellow Card scheme since its implementation. The objective is to describe temporal changes (in trends) in monthly antimicrobial consumption at pig-farm level from 2010 to 2017 after the implementation of the Yellow Card Scheme.

## Materials and Methods

In this study, a farm is defined as a single location with its unique identifier in the Central Husbandry Register (CHR), the CHR number. Each age group within a farm is individually referred to as “a herd” throughout the manuscript.

### Data Sources

Two databases were used for estimating the antimicrobial usage per animal on the farm level: the Vetstat database and the “Manure and Husbandry Database” (Manure Account).

The Manure Account is a national database into which the farmers account for all production of animal manure and use of manure on Danish farmland (hobby husbandry excepted). This includes exact data on the number of animals stabled or produced within specific age groups, including animal weights at entrance and exit from active farms for the juvenile age groups. Data for 2010 to 2017 were provided by the authorities (Landbrugsstyrelsen) for this project, while data for 2008 and 2009 are not stored in the database. From these data, animal biomass was estimated for each herd (the three age categories: sows, weaners, and finishers), for each year from 2010 to 2017.

### Antimicrobial Usage

All purchases of antimicrobials made between January 2008 and December 2017 for Danish pig farms were extracted from VetStat to be included in the analysis. Each record comprises the date of purchase, CHR number, target species, age group, and disease group at the time of prescription, and detailed information on the prescribed medicines. Internal validation showed inconsistency between recorded species in the CHR and recorded species in VetStat in <1% of records for the included farms. These errors were corrected when possible, based on a comparison of species registered on the farm and information on the prescription record (which included species, age group, and medicinal product). The data were further cleaned by deleting the negative records of antimicrobial purchases along with their corresponding positive records because of a lag of weeks or even months occasionally occurs. Negative records occur when prescribed medicines are not collected at the pharmacy.

The unit of measurement for antimicrobial usage used in this study was the Animal Daily Doses (ADD) defined (in VetStat) by the Food Administration in 2017 (9, 10). An ADD defines, for a given medicinal product, the average approved dose of treatment (in mg/kg body weight) multiplied with standard bodyweight for the age group in question. The standard bodyweight is the assumed average weight at treatment within the age group. Thus, the ADD is a standardized measure of amounts of antimicrobials, but only a rough estimate of the number of treatments.

The monthly amount of antimicrobials consumed per 100 animals within a herd (monthly number of ADD/(100 pig^*^days) at herd level) was calculated for each herd on each farm, in the following two steps:

1. For each date, the average daily use of antimicrobials within the herd was estimated, as the number of ADD's prescribed divided by the number of days until the following prescription. The estimation procedure was based on the assumption that all purchased antimicrobials were used at a constant rate between two consecutive purchases. However, it illegal to prescribe medicine for more than 30 days in most herds (leftover medicine has to be prescribed once again by the veterinarian). When the time between two consecutive purchases was over 90 days, the prescribed medicine was assumed to be used at a constant rate within 90 days of prescription; for each date, the average daily use was calculated by dividing the amount purchased by 90 days.
2. The daily antimicrobial use data were then aggregated per month and divided by the estimated number of pigs in each herd (for the given calendar year) and by the number of days in the month, thus calculating the amount of antimicrobial usage per pig-day for each herd. For 2008 and 2009, the estimated numbers of animals per herd for 2010 were used, as Manure Account data for 2008 and 2009 were not available.

### Inclusion Criteria

For a herd to be included in the study, it has to fulfill all of the following criteria:

1. it should have an estimated (averaged) antimicrobial consumption at least for all months from January 2008 to June 2010;
2. it should have no longer than 6 consecutive months without any estimated antimicrobial consumption for weaners and no longer than 12 consecutive months without any estimated antimicrobial consumption for sows and finishers and be considered as an active herd according to manure account data; and
3. herds with unrealistic values for antimicrobial consumption were omitted from the dataset herds, corresponding to antimicrobial consumption ≥300 ADD/(100 pig^*^days) at herd level for finishers and weaners and ≥110 ADD/(100 pig^*^days) at herd level for sow herd were deleted.

These criteria imply that herds are excluded it they have prolonged periods without antimicrobial usage (e.g., organic herds or herds with extremely low consumption), as well as herds closed down in a long period; the latter comprise satellite herds which only produce animals when justified by the price of the meat.

Three subsets (categories) were created based on the antimicrobial consumption in 2008 and 2009 for each herd type: low (<median), moderate (≥median, <3Q), and high (≥3Q) consumption. As an exploratory analysis, each subset was tested for seasonality based on autocorrelation plots (ACF function in R) of individual time-series. Neither autocorrelation nor seasonality was found in these data.

### Modeling and Parameterization

A univariate dynamic linear model (DLM) with a local linear trend component, as described in detail by West and Harrison (11) and applied in previous studies (12–15), was used to model data at the herd level. A previous study showed that Bayesian forecasting methods adapt faster to changes in the data, compared to the deterministic Holt's linear trend methods for monitoring trends of time-series (14). The general aim of a DLM is to estimate the underlying true value of a given variable, which is expected to change over time. This is achieved by sequentially applying a Bayesian framework in which the observed data (i.e., antimicrobial consumption) are combined with available prior information in the previous time step *t*−1 and used to calculate a conditional distribution at a given time *t*. This condition distribution and the observed data for the given time *t* are used to calculate the underlying true value, also referred to as filtered mean value.

Briefly, the DLM is defined by a set of two equations, namely the observation equation (Equation 1) and the system equation (Equation 2):

(1) $$\begin{array}{rcl} Y_{t} & = & {\text{F}}^{\prime }\theta_{t}+v_{t},\;v_{t}\;\sim \;N\left(0,V\right) \end{array}$$

(2) $$\begin{array}{rcl} \theta_{t} & = & \text{G}\theta_{t-1}+{\text{w}}_{t},w_{t}\;\sim \;N\left(0,{\text{W}}_{t}\right) \end{array}$$

where Equation (1) describes how the observed values depend on the underlying parameter vector (θ_*t*_) and Equation (2) describes the systematic evolution of the parameter vector from time *t*−1 to *t*. The variance components (*V* and **W**_*t*_) are referred to as the observational variance and system variance, respectively. The observational variance (*V*) was kept constant, while the system variance (**W**_*t*_) was estimated continuously. The system variance was approximated using a discount factor (δ) which expresses the decay of information. The transposed design matrix (**F**′) had the following structure:

(3) $$\begin{array}{r} \text{F}\prime =\left[\begin{array}{cc} 1 & 0 \end{array}\right], \end{array}$$

while the system matrix (**G**) was given the following structure:

(4) $$\begin{array}{r} \text{G}=\left[\begin{array}{cc} 1 & 1\\ 0 & 1 \end{array}\right] \end{array}$$

The model included a linear growth component consisting of a local linear trend defined by making the parameter vector a column vector with a length of 2:

(5) $$\begin{array}{r} \theta_{t}=\left[\begin{array}{c} m_{t}\\ T_{t} \end{array}\right], \end{array}$$

where *m*_*t*_ is the filtered mean of the antimicrobial consumption at time *t* and *T*_*t*_ is the local linear trend at time *t*. This local linear trend was incorporated into the model to allow the system to adapt to a possible positive or negative growth in antimicrobial consumption as follows the example given in previous studies (13–15).

A detailed description of the full model, as well as the R code, can be found in the literature (15).

The adaptive coefficient (calculated as part of the Kalman filter) and the filtered local linear trend values obtained from the DLM for the different datasets were visually assessed to determine the burn-in period of the model.

### Data Analysis

Data from 2008 and 2009 were used to define the initial prior distributions and the variance components (δ and *V*) of the models. Different combinations of values for *V* and δ were tested to optimize the models. This was done to ensure that the models would be optimized for forecasting the data from 2010 and onwards. Values of *V* ranging from 1 to 670 in increments of 0.1 were evaluated. Similarly, values of δ ranging from 0.1 to 1 by steps of 0.01 were tested. The combination of δ and *V* that minimized the sum of the squared forecast errors, and where the standardized forecast most closely followed a standard normal distribution, was chosen as the final variance parameters of the respective models. Deviations between forecasted and observed values are an indication that the herds were changing their antimicrobial consumption compared to their consumption in the past. The models were then applied to data until the end of 2017 to estimate investigate temporal changes on antimicrobial consumption for the different herds and compare them with changes in the Yellow Card scheme legislation. The nine different data sets (i.e., individual time series with data corresponding to weaner, sow, and finisher herds with low, high, and moderate antimicrobial consumption in 2008 and 2009) were included in the analysis and assumed to be mutually independent.

### Monitoring Changes in Data Streams

The growth was extracted from the θ vector of the model for each time step *t* for every single herd. The variance of the trend parameter, calculated from the variance-covariance matrix for the posterior distribution, as previously described (12), was used to calculate 95% credible intervals (CI). The percentage of herds with a negative growth or a significant decline (based on 95%) below zero, CI in the antimicrobial consumption for a given month *i* were both calculated as:

(6) $$\begin{array}{r} {\text{\% of herds }}_{i}=\;\frac{\;\sum x_{i}}{{n.herd}_{i}}\cdot 100\;\% \end{array}$$

where *x*_*i*_ is the number of herds with a negative growth or a significant decline for a given month *i* and *n.herd*_*i*_ corresponds to the total number of active herds (i.e., herds with antimicrobial consumption data) for a month *i*.

All data management and analysis were carried out in R (version 3.3.3).

### Key Dates Related to Introduction and Changes in the Yellow Card Scheme Legislation

The decision to implement the Yellow Card scheme was taken by the minister by January 2010, in response to the increasing antimicrobial usage in 2009 and earlier years. In the second quarter of 2010, the Danish Agriculture and Meat Council (SEGES), representing the pig producers, was informed about the decision and the upcoming Yellow Card. On June 30 and the first week of July 2010, letters were sent out to all pig producers with antimicrobial consumption over or close to the first Yellow Card limits, warning about the risk of receiving a Yellow Card, but also providing a chance to correct erroneous records in the databases. The dates when the legislation for the Yellow Card scheme and later changes were announced and implemented are described in Table 1.

**Table 1:** Changes in the Yellow Card scheme legislation in Denmark since its implementation.

**The publication date of new legislation**	**The period when Yellow Cards were issued based on the new thresholds^b^**	**Piglets, sows, and gilts^a^**	**Weaners up to 30 kg^a^**	**Slaughter pigs and gilts^a^**
2 Dec 2010	Aug 2011–May 2013	5.2	28	8
31 Aug 2012	Jun 2013–Oct 2014	5	25	7
27 Feb 2014	Nov 2014–Mar 2017	4.3	22.9	5.9
29 Jun 2016	Apr 2017–Dec 2017	4.1	21.8	5.6
20 Dec 2016	Jan 2018–	3.8	20.2	5.2

a Thresholds for antimicrobial consumption measured in ADD/(100 pigs*days) at the herd level.

b *After the announcement of new legislation, Yellow Cards were issued according to the new thresholds from 9 months after the legislation date, i.e., based on antimicrobial usage forwards from date of the legislation*.

## Results

**Figure 1:**
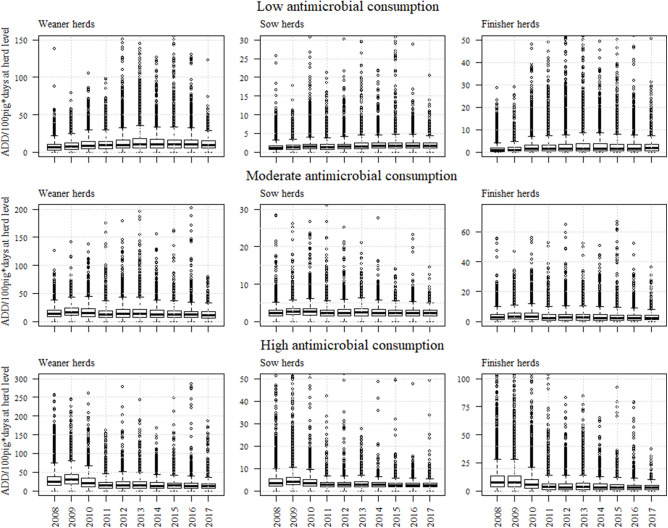
Boxplots with yearly antimicrobial consumption in weaners, sows, and finisher herds within different categories of consumption between 2008 and 2017. The medians obtained from the model are visualized more detailed in Figures 2–4. The categories were calculated based on data from 2008 and 2009 based on the median and on the 3rd quartile as thresholds.

A total of 2,310,160 purchases of antimicrobials made between January 2008 and December 2017 for 7,328 Danish pig farms were extracted from VetStat to be included in the analysis. This corresponded to a total of 3,513 weaner herds, 2,617 sow herds, and 6,496 finisher herds from which 1,538 weaner herds, 1,663 sow herds, and 1,999 finisher herds had an estimated (averaged) antimicrobial consumption data for at least all months between January 2008 and June 2010 (inclusion criteria no. 1). A total of 1,294 weaner herds, 1,633 sow herds, and 1,831 finisher herds had no longer than 6 or 12 consecutive months without any estimated antimicrobial consumption and be considered as an active herd according to manure account data (inclusion criteria no. 2). After removing herds with unrealistic values for antimicrobial consumption at herd level (inclusion criteria no. 3), a total of 1,282 weaner herds, 1,161 sow herds, and 1,828 finisher herds were included in the analysis. Table 2 describes the number of herds included in each category of antimicrobial consumption. The number of pigs in the study population was stable over the study period (Supplementary Material). In 2017, the study population comprised 67, 48, and 46% of the total number of sows, weaners, and finishers, respectably, in Denmark (population size estimated as described in DANMAP 2018). Figure 1 describes the yearly consumption of antimicrobials for the different herds between 2008 and 2017 in Danish pig herds.

**Table 2:** Number of herds included in the study and the corresponding cut-off values used to classify the herds regarding their antimicrobial consumption.

**Antimicrobial consumption^a^**	**Herd type**	**Cut-off**	**No. of herds**
Low	Weaner	<13.1	653
	Sow	<2.2	815
	Finisher	<2.9	912
Moderate	Weaner	≥13.1; <21.0	323
	Sow	≥2.2; <3.2	392
	Finisher	≥2.9; <5.2	477
High	Weaner	≥21.0	306
	Sow	≥3.2	409
	Finisher	≥5.2	439

a *Categories, calculated based on antimicrobial usage data from 2008 and 2009. The cut-offs were defined based on the median and on the 3rd quartile (3Q) of antimicrobial consumption*.

Table 3 describes the optimized values of the discount factors (δ) and observational variance (V) used for modeling the data. Based on visual inspection of the adaptive coefficient and growth components of the DLMs, we found that the models were stable against variations to the initial prior distribution, once the burn-in period of the models (14 observations) has been completed.

**Table 3:** Values for the discount factor (δ) and observational variance (V) obtained from antimicrobial consumption data between 2008 and 2009 for weaner, sow, and finisher herds.

**Antimicrobial consumption**	**Herd**	**Discount factor (δ)**	**Observational variance (*V*)**
Low	Weaner	0.84	34.0
	Sow	0.90	1.2
	Finisher	0.87	2.6
Moderate	Weaner	0.88	151.0
	Sow	0.96	2.0
	Finisher	0.93	9.0
High	Weaner	0.69	665.0
	Sow	0.83	11.0
	Finisher	0.67	115.0

**Figure 2:**
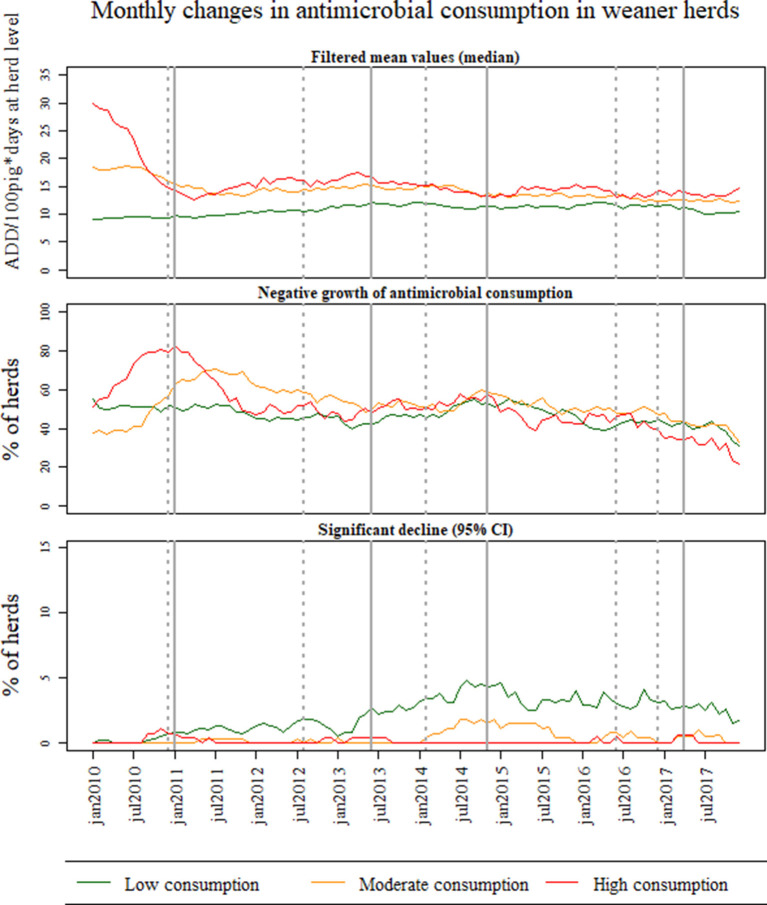
Monthly changes in antimicrobial consumption in Danish weaner herds between January 2010 and December 2017 for all Danish farms. The farms were divided into three groups based on the level of antimicrobial use (low, medium, and high) in 2008 and 2009. For each group of antimicrobial consumption, the graphs depict (1) the median of the filtered mean values obtained at farm-level based on the Dynamic Linear Model (DLM) with a linear trend component, (2) the percentage of herds with negative growth based on the DLM, and (3) the percentage of herds with a significant decline on the trend component of the DLM using 95% credible intervals (CI).

**Figure 3:**
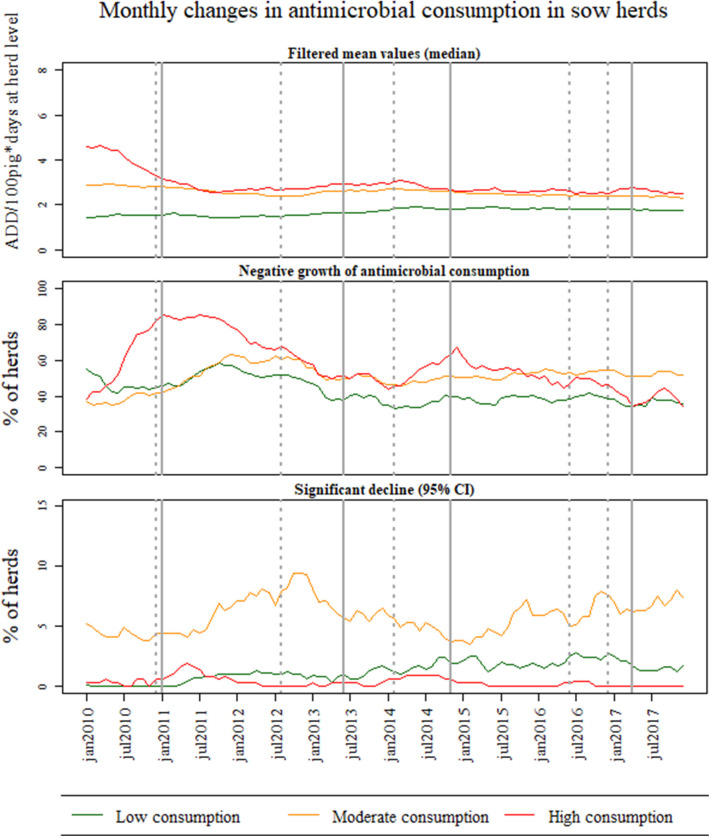
Monthly changes in antimicrobial consumption in Danish sow herds between January 2010 and December 2017 for all Danish farms. The farms were divided into three groups based on the level of antimicrobial use (low, medium, and high) in 2008 and 2009. For each group of antimicrobial consumption, the graphs depict (1) the median of the filtered mean values obtained at farm-level based on the Dynamic Linear Model (DLM) with a linear trend component, (2) the percentage of herds with negative growth based on the DLM, and (3) the percentage of herds with a significant decline on the trend component of the DLM using 95% credible intervals (CI).

**Figure 4:**
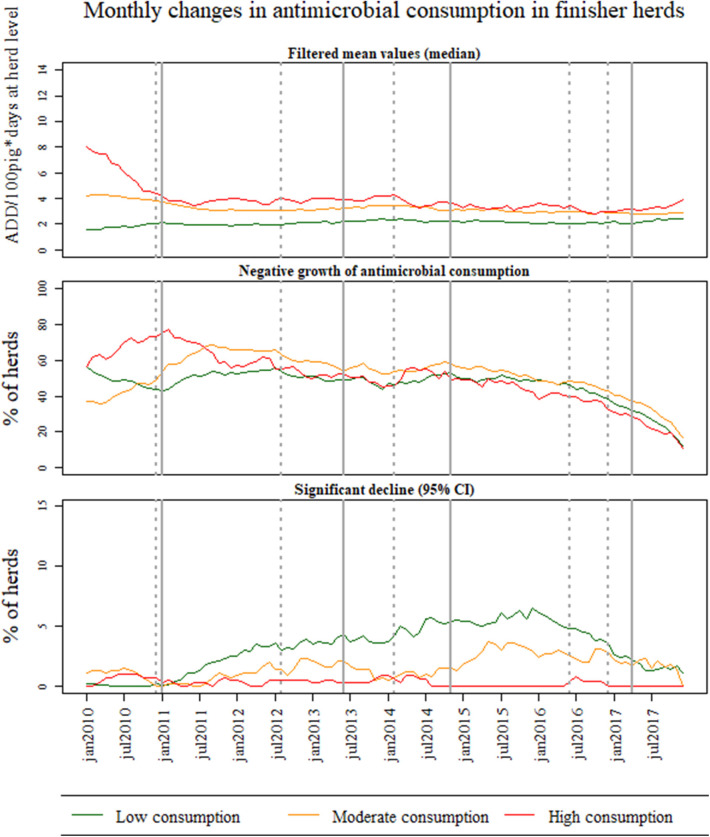
Monthly changes in antimicrobial consumption in Danish finisher herds between January 2010 and December 2017 for all Danish farms. The farms were divided into three groups based on the level of antimicrobial use (low, medium, and high) in 2008 and 2009. For each group of antimicrobial consumption, the graphs depict (1) the median of the filtered mean values obtained at farm-level based on the Dynamic Linear Model (DLM) with a linear trend component, (2) the percentage of herds with negative growth based on the DLM, and (3) the percentage of herds with a significant decline on the trend component of the DLM using 95% credible intervals (CI).

Figures 2–4 show the results of temporal changes in antimicrobial usage at pig-farm level; each figure shows (a) the median values of the filtered mean values obtained for each farm based on the DLM, (b) the percentage of herds with a negative growth, and (c) the percentage of herds with a significant decline in the growth using 95% CI.

Figure 2 shows the results of the temporal changes in antimicrobial usage in weaner herds at pig-farm level. The antimicrobial consumption in weaner herds with high antimicrobial consumption in 2008 and 2009 had a significant drop from the 2nd quarter of 2010 to the first quarter of 2011 of about 50% (Figure 2 based on the mean filtered values obtained from the DLM). This type of weaner herds had a higher percentage of herds with a negative growth component reaching 80% of herds during 2010, remaining above 60% until July 2011. Weaner herds with moderate antimicrobial consumption had a high percentage of herds with a negative growth form the end of 2010 and into the following years (Figure 2). Weaner herds with low antimicrobial consumption in 2008 and 2009 had the highest percentage of herds with significant declines (95% CI) in the growth component between 2011 and 2017 (as compared to the high and moderate consumption groups). However, the median consumption in this group was stable throughout the study period (Figure 2).

Figure 3 shows an overall decline of antimicrobial consumption between 2010 and 2017 in sow herds at pig-farm level. Similar to weaner herds, sow herds within the high antimicrobial consumption category presented a higher percentage of herds with a negative growth from 3rd quarter of 2010 and remained above 50% until the end of 2012 (i.e., during a prolonged period compared to the other herds in different categories of antimicrobial consumption). Noteworthy is the increase in the percentage of sow herds with negative growth within this category also in the second half of 2014. Sow herds with moderate antimicrobial consumption represented an increasing proportion of herds with negative growth from 2010, but with a lower rate compared to the high consumption category. For the moderate category, the highest proportion of negative growth occurred from the end of 2011 throughout 2012. Curiously, sow herds with moderate antimicrobial consumption represented the highest percentage of herds with a significant decline for the entire study period.

Figure 4 shows the changes in antimicrobial consumption for finisher herds at pig-farm level. Similar to weaner herds, the filtered mean values obtained from the DLM in the high consumption group of finisher herds decreased with about 50% from 2010 to the second quarter of 2011, with an increase in negative growth in the same period. For finisher herds in the moderate usage category, the percentage with negative growth increased from 38% in January 2010 to 71% in July 2011. Interestingly, there is a clear decrease in antimicrobial consumption (high consumption category) after January 2014, in the same period as new legislation was announced concurrently with changes in the ADD. In this period, an increase occurred in the number of finisher herds with significant declines, and a clear increase in negative growth was also seen among herds in the high consumption group. For all three herd categories of antimicrobial consumption, there was a constant decline in the percentage of finisher herds with negative growth after November 2014. Nevertheless, the median (Figure 4), as well as the mean antimicrobial usage, was at a stable level in all three groups during this period (Supplementary Material).

## Discussion

This study describes the dynamics of antimicrobial usage in Danish pig herds in reaction to the introduction and changes in the Yellow Card scheme.

As herds with very low, non-use, or intermitting usage are excluded, any trends in those herds are not described. However, the study sample is large, including the two-thirds of the sow population and almost half of the finisher and weaner population, and is most likely more representative for herds with moderate to high consumption due to the selection criteria. Therefore, as the Yellow Card is directed toward herds with moderate to high antimicrobial usage, the observations in this study may be considered representative of the general effects of the Yellow Card.

Antimicrobial usage data were analyzed at herd-level (i.e., separate for age each group) due to physiological differences (e.g., differences in the immune system due to aging and vaccination protocols), as these result in different amounts of antimicrobials prescribed for the three age groups (8, 16). It is evident that the optimized values of the observational variance (V) for weaner herds with high consumption is six times higher when compared to finisher herds within the same category of antimicrobial consumption. This is an indication that the antimicrobial consumption is higher and fluctuates more over time and between weaner herds when compared to sows and finisher herds due to physiological differences. For the same reason, the variance is higher in the high consumption categories, compared to the moderate, again over the low consumption categories.

Outliers with extremely high consumption estimates were excluded because they were likely due to data errors. In principle, animals cannot be treated with more than one daily dose per day. For sows, the limit for exclusion was set at 110 ADD/(100 pig^*^days), because the average weight of a fully grown sow is about 10% higher than the defined standard body weights (set at 200 kg, which is based on a proportion of gilts within sow herds). For weaners and finishers, the limit was set at 300 ADD/(100 pig^*^days), due to two aspects; firstly, if an outbreak occurs at the end of a production circle, the body weight may be almost twice the standard weight for the age group, suggesting a limit at 200 ADD/(100 pig^*^days); additionally, the number of weaners and finishers are estimated from the yearly production statistics. If the number of animals fluctuates within a year (e.g., satellite herds that close several months per year or purchase and breeding), the number of animals would be underestimated for some months and overestimated in others. Therefore, the threshold was increased by additionally 50% in these age categories.

From statistics on the National level, there seems to be some seasonality in the antimicrobial usage in pig production (8, 17). However, in this study, no seasonality was observed. One explanation could be that herds with very low consumption and herds that are closed part of the year were excluded. It seems likely, that the seasonality in antimicrobial usage may be due to a seasonal occurrence of disease in herds with a generally low antimicrobial usage.

In this study, the ADDs as calculated in 2017 were used across the entire study period. This was done to standardize the measurement of antimicrobial use and to avoid bias from changes in ADDs over time. Before 2014, the ADDs were defined for each product, based on the recommended values, and equivalent products could have different ADD's measured in mg/kg animal (different weights). Consequently, it was possible for the veterinarian to lower the estimated antimicrobial use in a herd as calculated by the authorities, simply by choosing a different brand. In 2011 and 2013, the prescription pattern changed toward products that had less weight in the Yellow Card scheme (data not published). Therefore, in 2014, the definition of the ADD's was changed, so that the ADD (mg/kg) has since been the same for all products with the same active compound and administration route.

The herds were categorized based on the antimicrobial usage in 2008 and 2009, and they remain in the same category throughout the study even though the individual herd may have changed the level of antimicrobial usage. This should be kept in mind when interpreting the data, especially late in the study period, when antimicrobial usage level may have changed dramatically in many herds. Thus, antimicrobial usage converges between the three categories of antimicrobial consumption over time. This is most important for the moderate and high consumption groups, where the antimicrobial used (median) has declined importantly over time, also with convergence between the two groups. Regarding the low consumption groups, the results suggest that the vast majority of herds in this category remained at a low level: firstly, the medians were stable throughout the study period and secondly, the negative growth curve remains around or just below 50%, showing that the number of herds with increasing and decreasing use is almost the same the entire study period (balanced fluctuation). Thus, the mean antimicrobial consumption across the herds within the low consumption categories increased only slightly throughout the study period and remained well below the other groups (Supplemental Material). The median for the high consumption category approached the median for the moderate consumption category, for all three age groups, and these two groups were very similar after 2013, with few exceptions. A similar pattern is seen regarding the mean consumption in these groups. However, for the finishers, the mean (and not the median) antimicrobial usage remained higher in the high consumption group compared to the moderate group for a longer period. This suggests that for finishers, the consumption has remained high in a proportion of the finisher herds. One explanation for this could be that the denominator used in the Yellow Card scheme is the number of animals registered in the CHR, and for many herds, the number of finishers is severely overestimated in the CHR. This allows for the maintenance of a high consumption level in some herds without risk of receiving a Yellow Card.

The results evidence a decline of the antimicrobial consumption in Danish pig herds more pronounced during two periods: the introduction of the Yellow Card, and in relation to the announcements and changes in the legislation in 2014. In general, herds with high antimicrobial consumption presented to have a steep drop (both mean and median) on antimicrobial usage from the 2nd quarter of 2010; this coincides with when the warning letters were sent out to the farmers with the highest consumption, prior to the implementation of the Yellow Card. This was also associated with a large increase in the proportion of herds with negative growth in the high consumption groups for all three age groups. Furthermore, the percentage of herds within the moderate and low antimicrobial consumption categories had an increase in the proportion with negative growth of antimicrobial consumption during 2011, i.e., after the Yellow Card legislation was in place.

Our results show an increasing trend in antimicrobial use in 2012–2014, which could be explained, at least in part by the possibilities of increasing the use without increasing the risk of receiving a Yellow Card, by manipulation of the calculated antimicrobial use. This option stopped at the beginning of 2014, at the same time as major down-regulation of the Yellow Card thresholds were published. These were the most prominent changes in the Yellow Card threshold after 2011, which could explain the coinciding increases in negative growth and declines in the median consumption throughout 2014 for the high consumption groups, particularly finishers and sows.

After 2015 until the end of 2017, a declining trend in negative growth for all herds is observed, showing that more herds have an increasing use than the number of herds with decreasing use. Regarding the regulations in 2016–2017, the aim was mainly the implementation of different weights to specific antimicrobials (3), whereas the thresholds were only regulated marginally. It is therefore not surprising that no clear effects were observed regarding the median antimicrobial usage on herd level. Nevertheless, the mean antimicrobial usage was slightly lower in the moderate and high consumption groups of finishers and weaners in 2017 compared to 2016 (Supplementary Material).

For herds with low antimicrobial consumption in 2008 and 2009, the median and mean antimicrobial use did not decline during the study period, rather small increases were observed. Nevertheless, both weaners and finisher herd in the low category had a higher percentage of herds with significant declines (based on 95% CI) in the growth component for longer periods of time when compared to any of the other categories of antimicrobial consumption. A likely explanation for this is the lower variance in the data and, consequently, narrowed CI rather than larger decreases on antimicrobial consumption. The declines in the other categories of antimicrobial consumption are higher but, due to larger CI, the percentage of herds with significant declines is lower.

For sows, the percentage of herds with significant declines in the group of moderate antimicrobial use was much higher than for the other groups. This reflects that the variance is similar to the variance in the low consumption group, but as the consumption is much higher it left more room for reductions. The percentage of herds with a significant decline is at about 5–10% throughout the entire study period. This means that every month 5–10% of the herds have significant declines. As the median is stable (with a slightly decreasing trend) for this group of sow herds, this indicates that the antimicrobial usage fluctuates very much in this group of sow herds.

## Conclusions

Our findings suggest that changes in the Yellow card legislation have had a major impact on the decreasing quantitative level of antimicrobial use in Danish pig herds in relation to regulations of the thresholds, mainly in pig herds with high antimicrobial usage. The changes in the legislation of the Yellow card scheme made by the Danish Food and Veterinary Administration have thus promoted a reduction in antimicrobial usage over close to a decade in pig farms.

## Data Availability Statement

Data cannot be shared publicly because of legal restrictions on sharing the identified dataset due to sensitive information collected at the farm level. The Danish Veterinary and Food Administration owns the antimicrobial consumption datasets used and analyzed in the current study. Data requests should be sent to the Danish Veterinary and Food Administrations (using the following link: https://www.foedevarestyrelsen.dk/english/Aboutus/Contact/Pages/default.aspx).

## Author Contributions

AL contributed to the study design, data management, and analysis and wrote the manuscript. VJ contributed to the study design, data management, and reviewed the manuscript.

### Conflict of Interest

The authors declare that the research was conducted in the absence of any commercial or financial relationships that could be construed as a potential conflict of interest.
