# Corrigendum: Close to a Decade of Decrease in Antimicrobial Usage in Danish Pig Production–Evaluating the Effect of the Yellow Card Scheme

**DOI:** 10.3389/fvets.2020.00469

**Published:** 2020-07-29

**Authors:** Ana Carolina Lopes Antunes, Vibeke Frøkjær Jensen

**Affiliations:** Division for Diagnostics & Scientific Advice—Epidemiology, Center for Diagnostics, National Veterinary Institute, Technical University of Denmark, Lyngby, Denmark

**Keywords:** antimicrobials, Yellow Card scheme, legislation, pig, Denmark

In the original article, there was a typographic error in the introduction regarding the human population and number of pigs in Denmark.

The text should read:

“In 2017 (3rd quarter), Denmark had 3,226 pig farms with 12.69 million animals (5) compared to a human population of 5.76 million individuals (6).”

The authors apologize for this error and state that this does not change the scientific conclusions of the article in any way. The original article has been updated.

