# Supplementary material for: Close to a Decade of Decrease in Antimicrobial Usage in Danish Pig Production–Evaluating the Effect of the Yellow Card Scheme
Source: Front Vet Sci. 2020 Mar 6;7:109. doi: 10.3389/fvets.2020.00109 (PMC7067903; doi:10.3389/fvets.2020.00109)
Supplement: Supplementary file 1 [file Data_Sheet_1.PDF]

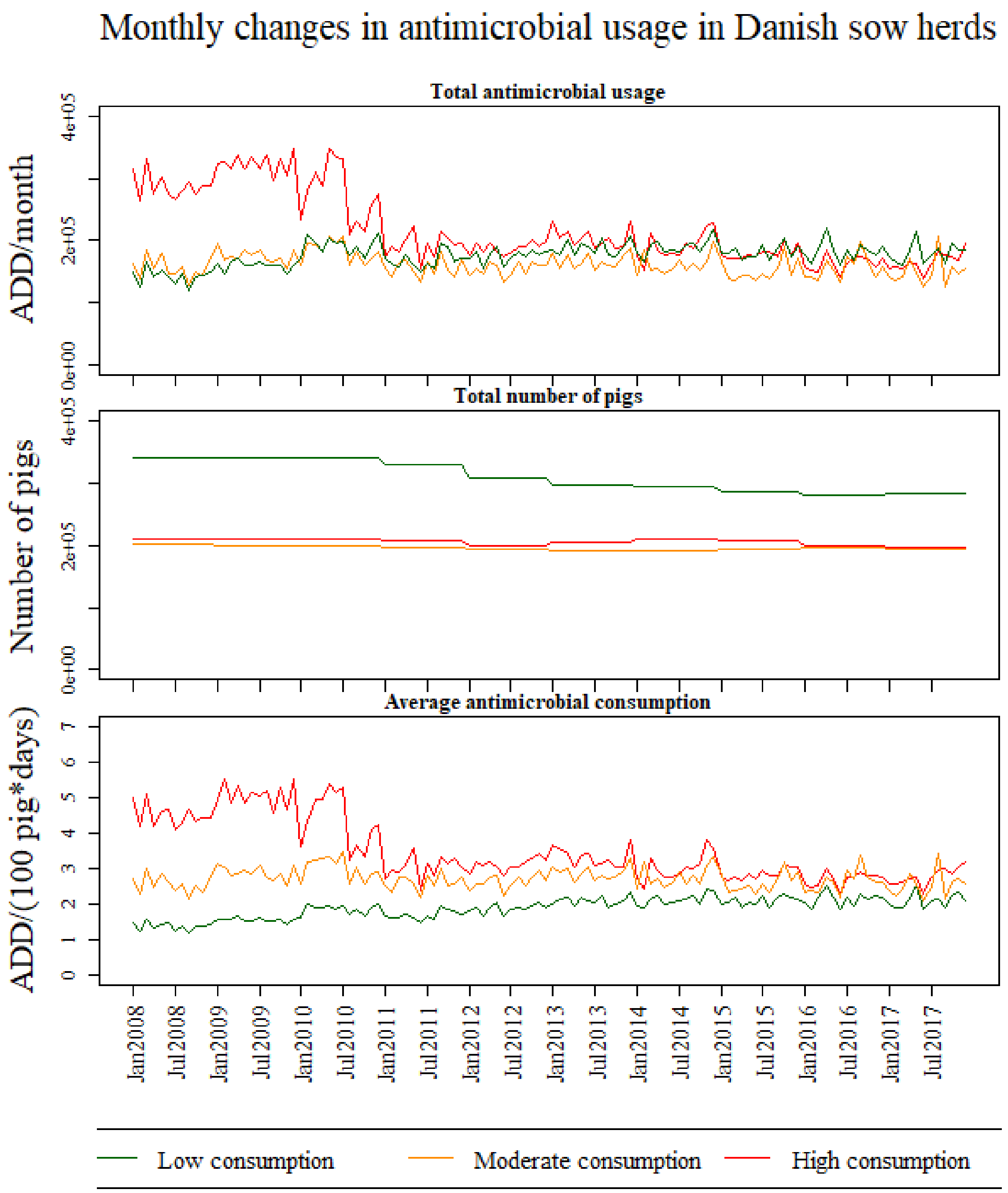

The categories (subpopulations) for the antimicrobial consumption were estimated based on data from 2008 and 2009 based on median and 3rd quartile values.
Total monthly antimicrobial consumption for the study subpopulations of sows was estimated from VetStat data.
Number of sows in the study subpopulations was estimated as a yearly average based on Manure Account data for each herd.
Monthly mean was calculated across each study subpopulation.

Monthly changes in antimicrobial usage in Danish weaner herd

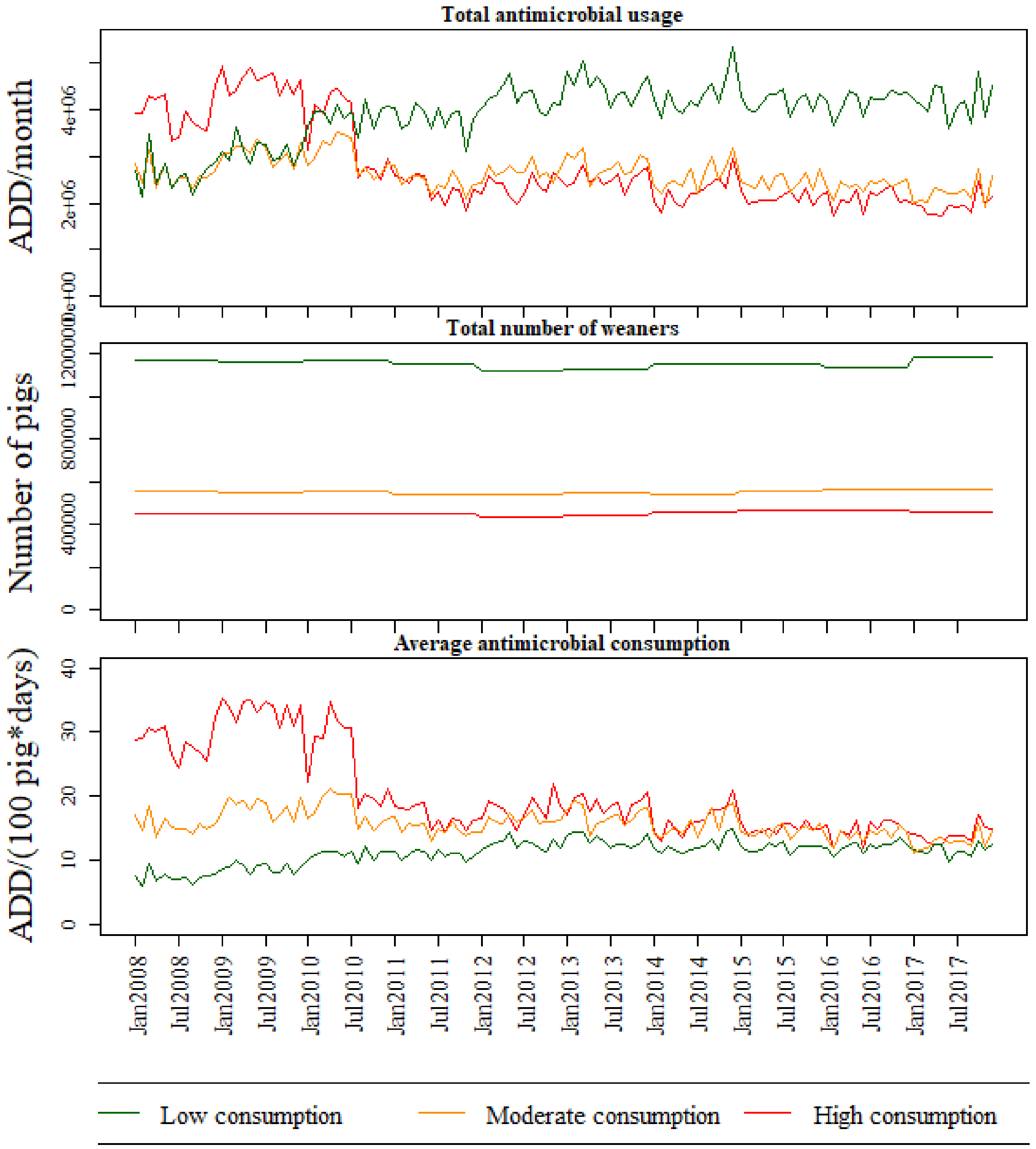

The categories (subpopulations) for the antimicrobial consumption were estimated based on data from 2008 and 2009 based on median and 3rd quartile values.
Total monthly antimicrobial consumption for the study subpopulations of weaners was estimated from VetStat data.
Number of weaners in the study subpopulations was estimated as a yearly average based on Manure Account data for each herd.
Monthly mean was calculated across each study subpopulation.

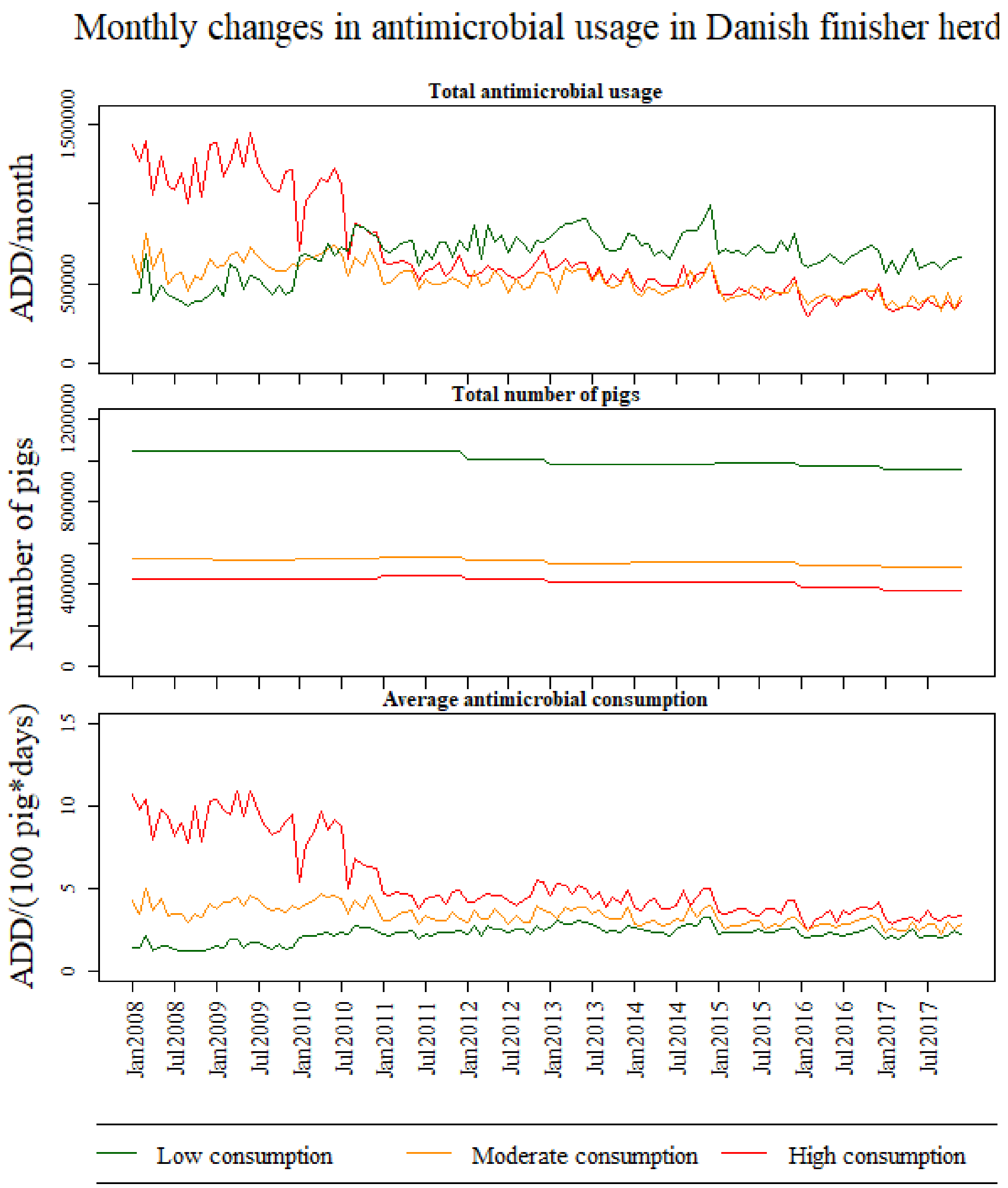

The categories (subpopulations) for the antimicrobial consumption were estimated based on data from 2008 and 2009 based on median and 3rd quartile values.
Total monthly antimicrobial consumption for the study subpopulations of finishers was estimated from VetStat data.
Number of finishers in the study subpopulations was estimated as a yearly average based on Manure Account data for each herd.
Monthly mean was calculated across each study subpopulation.
